# Effects of silencing Rab27a gene on biological characteristics and chemosensitivity of non-small cell lung cancer

**DOI:** 10.18632/oncotarget.21782

**Published:** 2017-10-10

**Authors:** Xia Li, Haiying Wang, Qinggan Ni, Zhiyuan Tang, Jun Ni, Liqin Xu, Hua Huang, Songshi Ni, Jian Feng

**Affiliations:** ^1^ Department of Respiratory, Affiliated Hospital of Nantong University, Nantong 226001, Jiangsu, China; ^2^ Department of Rehabilitation, Affiliated Hospital of Nantong University, Nantong 226001, Jiangsu, China; ^3^ Department of Pathology, Affiliated Hospital of Nantong University, Nantong 226001, Jiangsu, China; ^4^ Department of Respiratory, Yancheng Third People’s Hospital, Yancheng 224002, Jiangsu, China; ^5^ Department of Central Laboratory, Affiliated Hospital of Nantong University, Nantong 226001, Jiangsu, China

**Keywords:** non-small cell lung cancer, Rab27a, biological characteristics, proliferation, migration

## Abstract

Rab27a, a member of the Rab protein family, can regulate the tumor microenvironment and promote the development of the tumor. Elevated expression of Rab27a is closely connected with many human cancers containing non-small cell lung cancer (NSCLC). But the role of Rab27a in non-small cell lung cancer and its possible mechanism is particularly unclear. In this research, we explored the effect of silencing Rab27a *in vitro* and *in vivo*, furnishing evidence that Rab27a could be a potential therapeutic target in NSCLC. Compared with corresponding control cells, silencing Rab27a had decreased ability of cell proliferation, migration and invasion *in vitro* and slower growth of xenograft tumors in mice. The expressions of apoptosis-associated proteins were induced with a reduction of anti-apoptotic protein in the NSCLC cells down-regulated Rab27a. Furthermore, Rab27a was associated with resistance to conventional chemotherapeutic agents. Our findings suggested that Rab27a might play a critical role in increasing chemosensitivity in NSCLC.

## INTRODUCTION

Lung cancer is the most common respiratory tract malignancy with the top incidence and mortality. A variety of factors contribute to the occurrence and development of lung cancer. The death rate of lung cancer in developed countries continues to hold the top spot, while in developing countries that is also increasing year by year. Depending on the histology, stage of lung cancer, and general condition of the patients, the principles of multi-modality treatment including surgery, irradiation, chemotherapy, as well as bio-therapy and gene therapy are extensively discussed [1–3]. Even so, cancer metastasis and drug resistance after systematic treatment is still a major problem for patients with lung cancer [4]. Prognosis of patients with lung cancer is ominous, with a 5-year survival rate less than 15% [5].Therefore, recent researchs of lung cancer are mainly concentrated on the identification of biomarkers in patients and the development of molecularly targeted drugs [6].

shRNA (short hairpin RNA) is a expression vector in which double-stranded RNA degrades its homological mRNA and leads to sequence specific post-transcriptional gene silencing in an organism [7]. With the development of technology, silencing targeted genes associated with the progress of lung cancer is a new hope for patients with lung cancer [8].

The Rab family consists of more than 60 members and is widely expressed in mammals. The Rab27a family, molecular weight from 20 to 30 kDa, plays a significant role in endocytosis, cell secretion, growth and signal transduction. Rab27a, a member of the Rab family, can regulate the tumor microenvironment and promote tumor growth. Recently, some studies have found that Rab27a has a close relationship with many human cancers, Rab27a overexpression prefigures unfavourable prognosis in colorectal cancer [9–12]. Rab27a promotes the invasion and metastasis of human breast cancer cells [13]. However, the association between Rab27a and NSCLC is not very clear. We conducted the present research to thoroughly explore the function of Rab27a in NSCLC development.

Here in this article, in order to illustrate the relationship between Rab27a and NSCLC, we silenced Rab27a gene of NSCLC cells, further explored the changes in cell invasion, migration, proliferation and apoptosis *in vitro* and tumor model of human NSCLC cells in nude mice. Our intent is to search for potential targets related to NSCLC and thus to provide evidence for early diagnosis and treatment of NSCLC.

## RESULTS

### The expression of Rab27a associated in NSCLC

**Figure 1:**
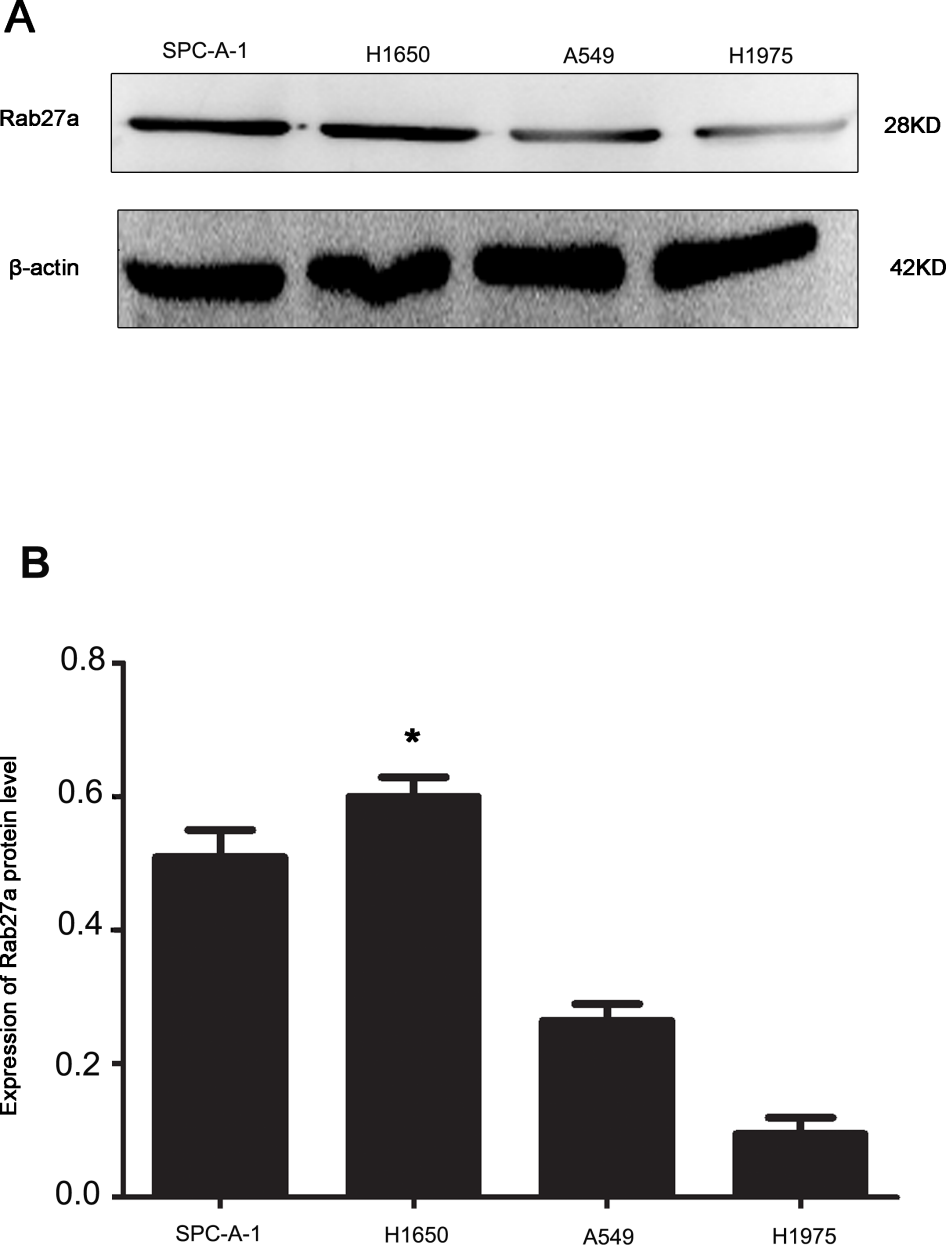
Expression of Rab27a protein in four non-small cell lung cancer cell lines **(A** and **B)** β-actin was used as loading control.

Our previous study showed Rab27a overexpression in the tissue of NSCLC. Here we tested Rab27a expression in four NSCLC lines (SPC-A-1, A549, H1650, H1975). Western blot results revealed that SPC-A-1 and H1650 cell lines expressed higher levels of Rab27a than H1975 and A549 cell lines (Figure 1A and 1B).

### The effect of silencing the expression of Rab27a on cell proliferation, migration and metastasis of NSCLC

**Figure 2:**
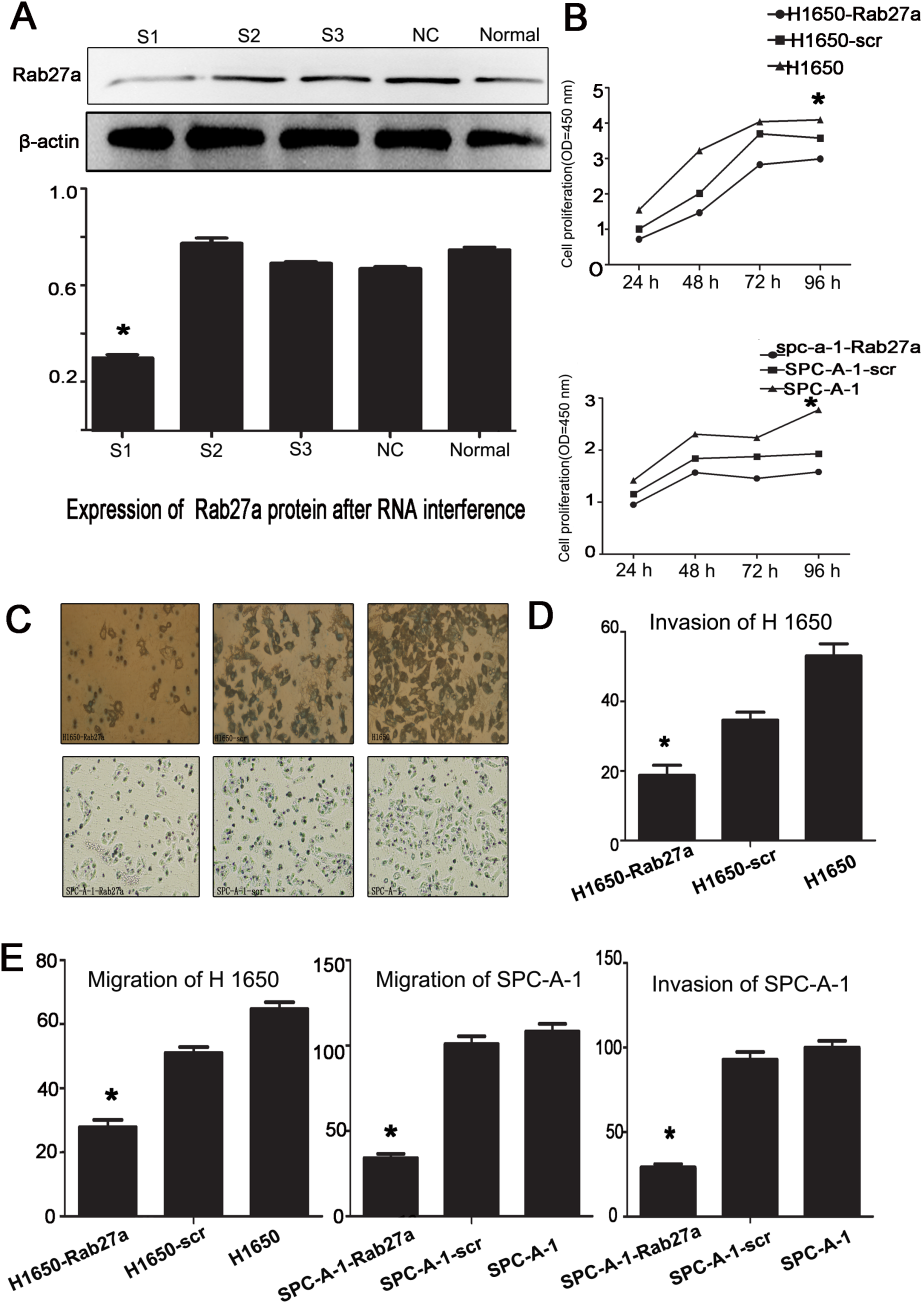
Effect of depleting or enforcing the expression of Rab27a on cell proliferation, migration and invasiveness of lung carcinoma cells **(A)** western blot were used to select the most effective silencing shRNA targeting human Rab27a. **(B)** The proliferation ability of the four experimental cell lines was examined using CCK-8 at 450 nm. Specifically, 5 × 10^3^ cells were seeded in 100 μL of medium per well into 96-well plates (three wells per each group). Then, 10 μL of CCK8 solution was added to the culture medium in each well after 24h, 48h, 72h and 96h. Then cells were incubated for another 3 h. The absorbance was determined at 450 nm wavelength. **(C-E)** Migration and invasion ability were presented as total number of cells that migrated to the bottom chamber without or with the transwell-precoated matrigel, respectively, as calculated in at least six random fields (total magnification ×200) per filter. (^*^, P<0.05).

Next we showed the functional of transfecting the Rab27a expressions in NSCLC cell lines by testing three different sequences of siRNA targeting Rab27a, negative control and without any intervention control siRNA. Western blot proved Rab27a#1 was the most convincing silent sequence (Figure 2A). We transfected the shRNA into H1650 and SPC-A-1 cell lines, for these two kinds of cell lines showed higher expression of Rab27a.

We next evaluated the ability of invasion, migration and proliferation in SPC-A-1 and H1650 cell lines. We found that after silenced with Rab27a shRNA (SPC-A-1-Rab27a and H1650-Rab27a) the proliferative capacity of cell lines was lower than the control groups (Figure 2B). In the experiment of invasion and migration, compared with the two control groups, fewer SPC-A-1-Rab27a and H1650-Rab27a cells migrated via the membrane in the migration chamber with or without the Transwell-precoated Matrigel, with statistical significance (Figure 2, C-E P < 0.05). The results of these experiments, silencing Rab27a expression can be effectively reduced the ability of proliferation, migration and invasion in NSCLC cells.

### Component activation and silencing of Rab27a on growth of NSCLC in nude mice

**Figure 3:**
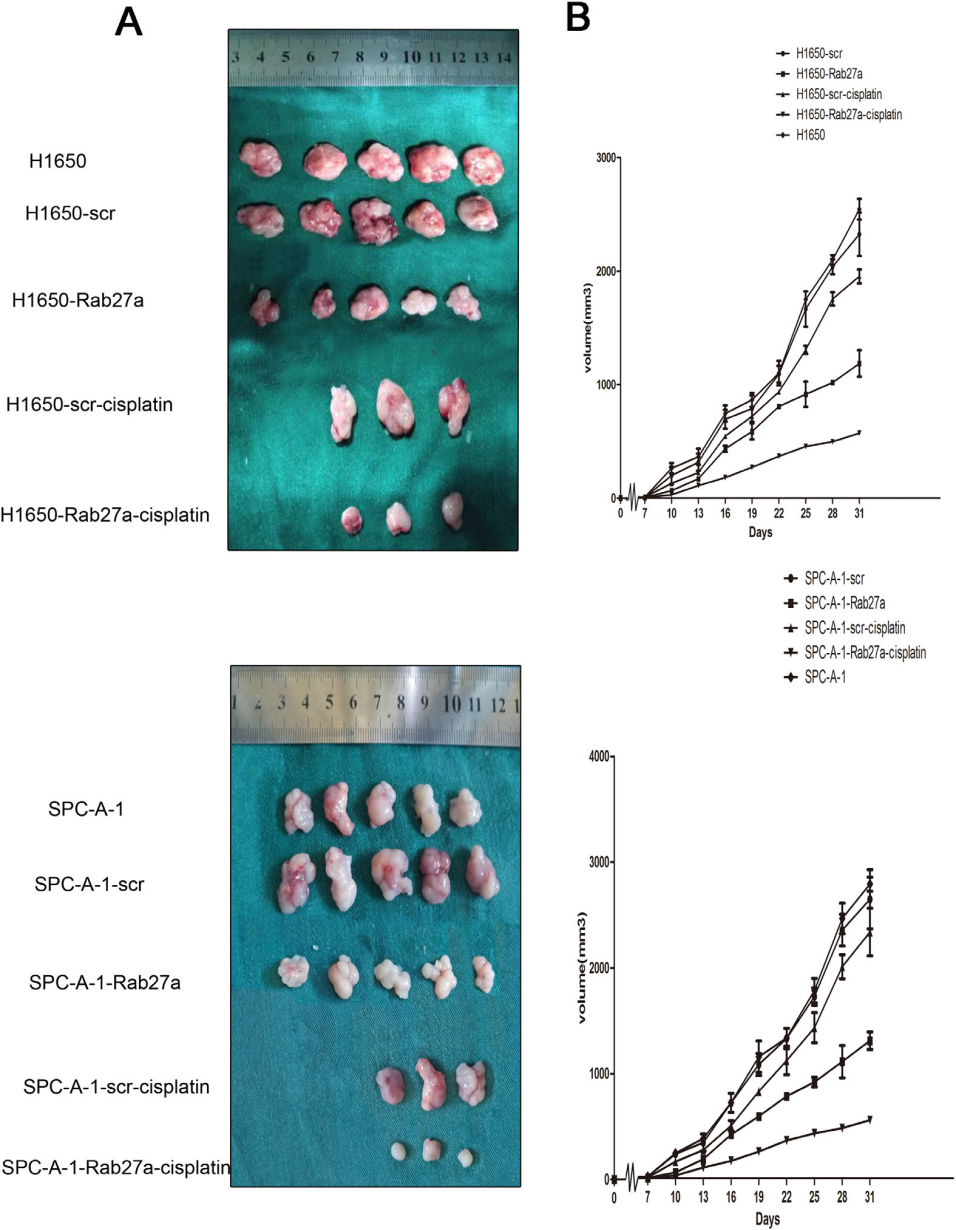
Effects of constitutive activation or silencing of Rab27a on growth of NSCLC in nude mice Experimental cells were subcutaneously injected into BALB/c nude mice. **(A)** Images of tumors in normal control group, Rab27a silenced groups, corresponding controls groups and including intraperitoneal injection of cisplatin groups. **(B)** The tumor size were measured every third day after inoculation, growth of tumors produced by subcutaneous injection of mice with H1650, H1650-scr, H1650-Rab27a, then H1650-scr, H1650-Rab27a treated by cisplatin. The tumors from mice that received cisplatin and stable transfection cell injection were smaller than those either treated with cisplatin or transfected alone. All date are shown as mean±SD. ^*^, *P*<0.05.

The effect of Rab27a on tumor growth was studied by injecting different cell lines into BALB/c athymic nude mice subcutaneous to construct xenografts model. We first examined tumor growth in negative control and normal control groups. There was no significant difference in tumor growth curve at each point in time (Figure 3A and 3B *P* > 0.05, A:*P*=0.436, B: *P*=0.728). Thereafter, cells in which the high expression of Rab27a was silenced (H1650-Rab27a and SPC-A-1-Rab27a), their accordant control cells (H1650-scr and SPC-A-1-scr) and normal control cells were injected into the sides of the lower edge of the ribs subcutaneous, and tumor size was measured and registered every three days. Tumor growth curves demonstrated that the H1650-scr and SPC-A-1-scr cells of tumor volume after silenced for Rab27a expression was much lower than corresponding control and normal control. The average tumor volume every mouse after injection of H1650-Rab27a cells was approximately 720 mm^3^ at 31 days. The average tumor volume every mouse after injection of H1650-scr cells was approximately was 2512 ± 172 mm^3^ at 31 days. There was significant difference between the two groups (P<0.05). Compared with the average tumor volume by SPC-A-1-scr (1985 ±182 mm^3^), the average tumor volume by SPC-A-1-Rab27a was reduced (562 ± 120 mm^3^) (P<0.05). Particularly, the tumor size of the mice that received stable transfection cell and cisplatin were significantly smaller than those only received one alone (*P*<0.05). Interestingly, we found an interesting phenomenon that tumor growth speed was significantly expedited at certain periods (22 d in H1650 and 25 d in SPC-A-1).

### Expression and apoptosis of the Rab27a *in vitro* and *vivo* experiments

**Figure 4:**
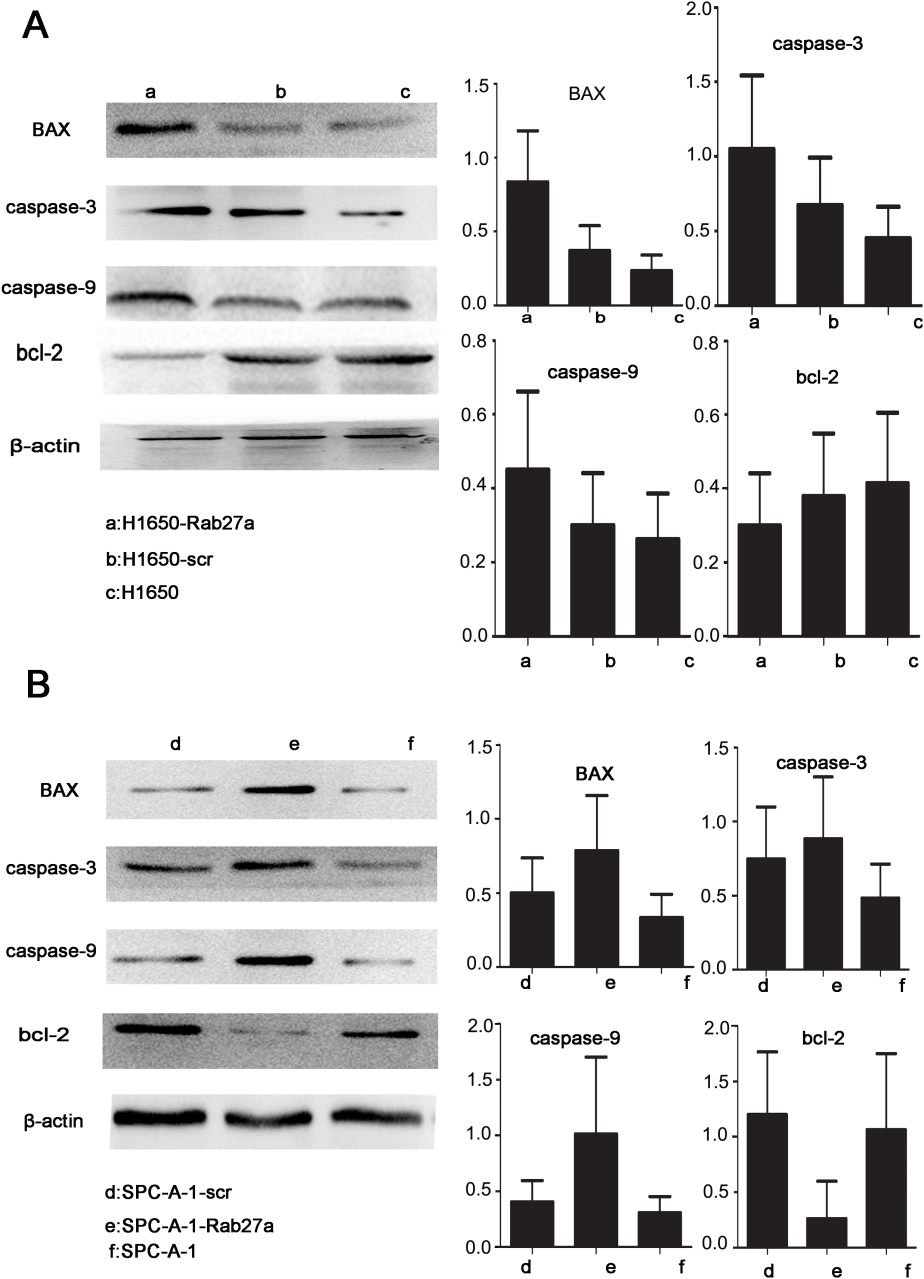
Rab27a expression and apoptosis *in vitro* **(A** and **B)** Western blot analysis of apoptosis-associated protein expression in Rab27a silenced cells and tissues. β-actin was used as a control.

By studying the expression of apoptosis protein after Rab27a silencing, the intrinsic mechanism of Rab27a induced growth of lung cancer cells was derived. Apoptotic protein caspase -3, caspe-9 and Bax expression were higher in spc-a-1-rab27a and h1650-rab27a cells than the corresponding control group and the normal control group. By comparison, the expression of anti-apoptotic protein Bcl-2 was reduced after Rab27a silencing,(Figure 4A and 4B). Those results indicated that silencing Rab27a expression could enhance apotosis in NSCLC cells, and that should be explored in future researches.

**Figure 5:**
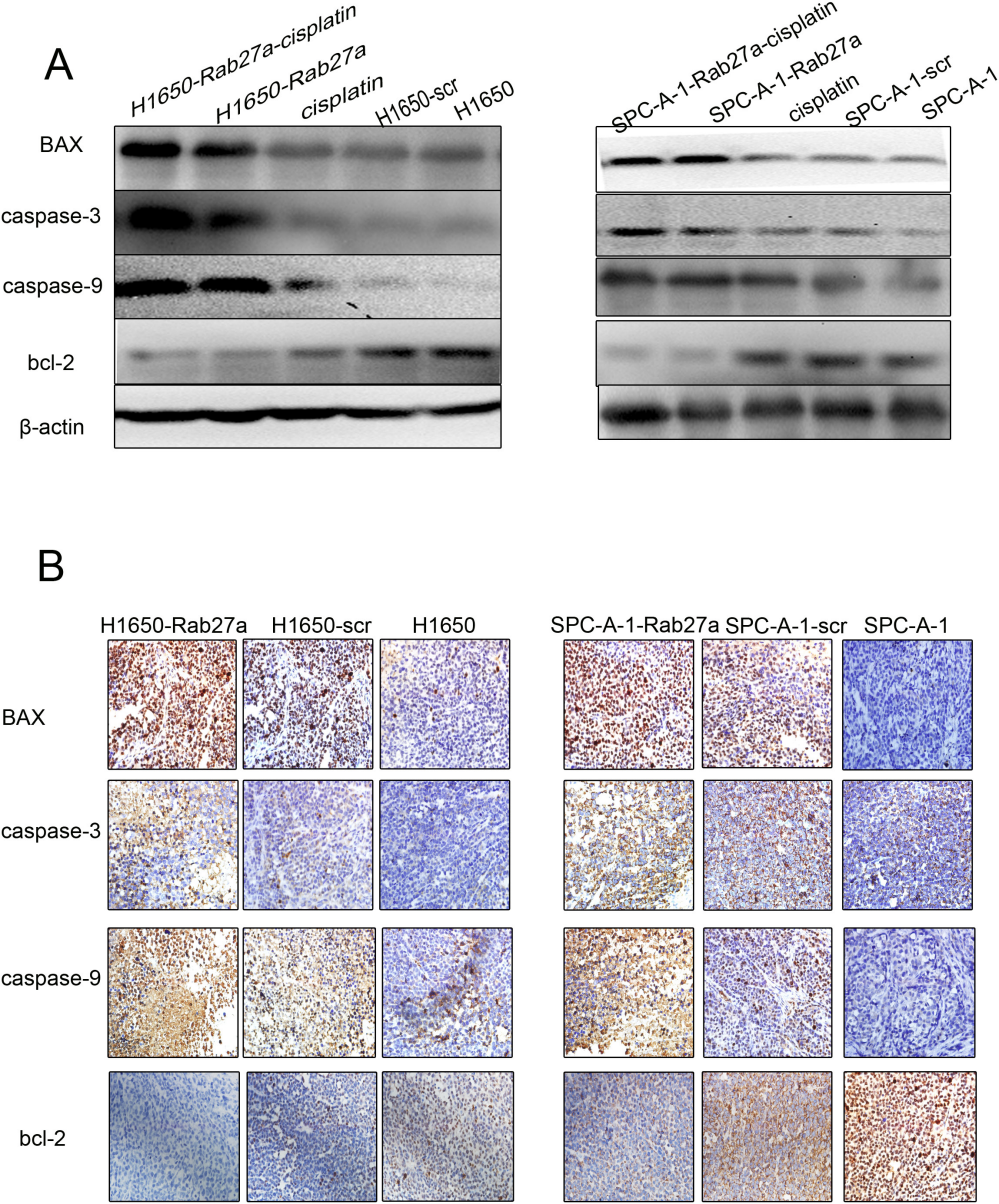
Rab27a expression and apoptosis *in vitro* **(A)** Western blot of apoptosisassociated protein expression in frozen tissues, including upon cisplatin combination treatment. β-actin was used as a loading control. **(B)** Analysis of apoptosis-associated protein expression on TMA was performed by immunohistochemistry staining (magnification ×200). The result was consistent with (A).

We injected cisplatin into three of the eight mice from Rab27a silenced groups by intraperitoneal injection. We found that the level of tumor tissue apoptosis was higher than the normal control group after cisplatin treatment, in addition, the combination of silencing and cisplatin treatment, the tumor tissue apoptosis level was significantly increased (Figure 5A). For immunohistochemistry staining, Bax, Caspase-3, Fas-L, Bcl-2 were tested on TMA. Observation of different levels of protein staining in the nucleus. The expression of Bax, caspase -3 and fasl was increased in h1650 - rab27a and h1650 - rab27a groups, and the expression of bcl - 2 was decreased. The number of apoptotic cells was the highest in shRNA and cisplatin combined tissues (Figure 5B). These results suggest that silencing Rab27a may positively regulate the apoptosis of NSCLC, whereas the use of cisplatin and targeting Rab27a binds more efficiently to promote apoptosis. We will continue to explore their mechanisms in future studies.

### Rab27a expression confers promoting chemotherapy-induced apoptosis

**Figure 6:**
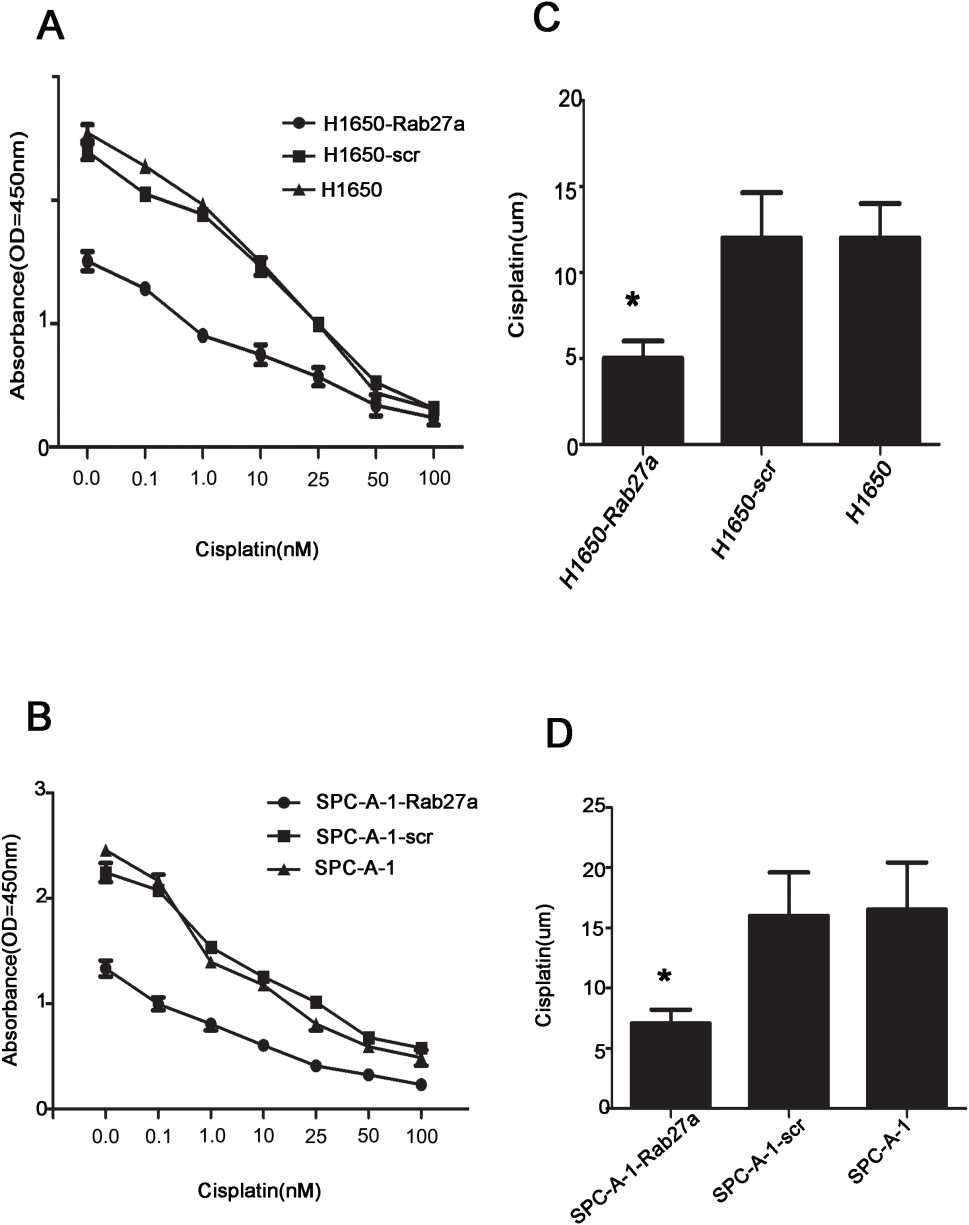
Rab27a expression increases sensitivity to chemotherapeutic drugs **(A-D)** Rab27a depletion in SPCA-1-Rab27a and H1650-Rab27a cells contributed to increased apoptotic response to cisplatin. (Error bars = 95% confidence interval, CI; P=0.003 and P=0.007 respectively).

24 hours after transfection, different concentrations of cisplatin were added in SPC-A-1-Rab27a and H1650-Rab27a cells, corresponding and normal controls cells, then we observed the three group cells by using the method named CCK8 after 48 hours and detected the changes of sensitivity in SPC-A-1 and H1650 cells to cisplatin. The results showed that the sensitivity of SPC-A-1-Rab27a and H1650-Rab27a cells to chemotherapeutic drugs increased significantly compared with the corresponding controls and normal controls (P < 0.05). It showed that the target Rab27a gene could increase drug sensitivity of lung cancer cells (Figure 6). We investigated the relationship between Rab27a expression and chemoresistance to four chemotherapeutic agents in NSCLC cells and examined whether Rab27a could regulate drug-induced apoptosis. The depletion of Rab27a in h1650 - Rab27a and SPC-A-1-Rab27a cells leads to an increased response to chemotherapy in NSCLC (Figure 6, A: P=0.004 and B: P=0.008). The IC50 in H1650-Rab27a and SPC-A-1-Rab27a cells ware lower than in control group cells (Figure 6C and 6D P<0.05). Thus, the downregulation of Rab27a can increase the sensitivity of NSCLC cells to chemotherapeutic agents, which may be related to above results.

## DISCUSSION

The occurrence of tumor is the result of the interaction of multiple genes. The main reason for the low survival rate of cancer patients is tumor metastasis and drug resistance. Tumor invasion of the surrounding tissue and the formation of distant metastases, resulting in other organ dysfunction and even organ failure, seriously affect the quality of life of patients and threaten the life of patients [14, 15]. The molecular mechanism of tumor invasion and metastasis has long been the focus of tumor research. Lung cancer is very common all over the world at present. Because of the invasion and metastasis, the recurrence rate is very high even in patients undergoing radical operation for lung cancer. And 70% of the patients diagnosed with lung cancer have been advanced, although several traditional chemotherapy regimens may prolong the survival of patients, many patients can not tolerate the side effects of chemotherapy [16–18]. So, finding new prognostic markers of lung cancer, exploring the mechanism of its partial mechanism, and taking targeted therapy are particularly important.

In recent years, significant progress has been made in the field of cancer genomics, especially in the occurrence and development of lung cancer, and the importance of epigenetic specificity has been widely recognized [19]. In the case that the nucleotide sequence of the gene is not changed, the epigenetic gene is a branch of genetics that expresses a genetic change. There are a lot of epigenetic phenomena, well-known as gene silencing, DNA methylation, nucleolar dominance, dormant transposon activation and genomic imprinting. Studying the changes of these epigenetic changes can identify some tumor related genes, and provide new insights and ideas for the treatment of lung cancer [20–22]. The topic chosen gene silencing technology, rapid and specific identification of target gene mRNA sequence, interference the protein synthesis, and down regulating the expression of target genes, obtaining target gene function loss or decreased expression, could achieve the purpose of disease treatment.

In this study, by RNA interference technology, we investigated the effects of Rab27a gene silencing on the biological characteristics of NSCLC and its role as a potential target for lung cancer treatment. At the cellular level, four human lung cancer cell lines were selected and screened by Western blot, and the high expression Rab27a cell lines were selected for subsequent silencing experiments. Western blot was applied to confirm the RNA interference technique that the gene silencing could affect the transcription and translation level thus reduce the expression of Rab27a, and the interference efficiency can reach 70%. Then we used the cell proliferation assay (CCK8 method) to find that the proliferative ability of lung cancer cells was obviously decreased after gene silencing; the ability of invasion and migration of lung cancer cells, confirmed by transwell migration and invasion assay, were significantly decreased after Rab27a gene interference. These biological effects of Rab27a in NSCLC were in accordance with those reported in other cancers (breast cancer [23, 24], pancreatic cancer [25], colorectal cancer [26, 27]and so on). In addition, through Western blot, we found that Rab27a gene interference cells highly expressed apoptosis related Caspase-3, Bax, caspase-9 expression compared with the control group, while the anti apoptotic protein bcl-2 expression was reduced, the differences were statistically significant (P < 0.05). In a word, reduction of Rab27a expression significantly promotes the apoptosis of lung cancer cells, resulting in inhibition of tumor growth.

We also studied the changes of the sensitivity to chemotherapy in cells after Rab27a gene silencing. We found that tumor shown in animals on injection of Rb27a knockout do not appear to be significantly lower in size or weight. The tumors are still big, therefore Rb27a may not be significant gene in regulation of tumor growth. However, treatment of animals bearing tumors generated by Rb27a knockout cells followed by cisplatin treatment show a significant reduction, therefore, Rb27a may be involved in changing the sensitivity of tumor cells for cisplatin, but whether directly involved in tumor growth is not clear. Therefore, the treatment focused on Rab27a gene could target multiple aspects of tumor treatment, for example, it could selectively increase the enzymatic activity of apoptosis gene and/or inhibit the expression of anti hole of apoptosis genes in tumor cells, strengthen the efficacy of chemotherapy or radiotherapy and reduce the toxic and side effects [28–30], thus provide a new reference for clinical chemotherapy drugs. So, it is extremely important to further explore the role of Rab27a in the regulation of NSCLC.

Our research results showed that in NSCLC cell level, interference gene Rab27a decreased significantly the proliferation and invasion of tumor, promoted apoptosis, and increased the sensitivity to chemotherapy drugs of NSCLC. Which provide experimental and theoretical basis for the treatment of lung cancer.

## MATERIALS AND METHODS

### Cell lines and cell culture

Human lung cancer cell lines A549, H1650, SPC-A-1 and H1975 were respectively purchased from the Shanghai Institute of Biochemistry and Cell biology, the Chinese Academy of Sciences. These four cell lines were cultured in medium (cyclone Chinese abstract, Logan City, Utah, USA) with 10% fetal bovine serum (FBS), 2 mm L-glutamine, 100 U/ml Penicillin/Streptomycin mixture (GIBCO BRL, Grand Island, NY, USA) under a humid atmosphere in 5% CO_2_ at 37°C.

### Western blot

Total protein of these tumour cells and tissues were extracted by a lysis buffer (Beyotime Institute of Biotechnology, Nantong, China), and protein concentration were measured by the BSA method (Beyotime Institute of Biotechnology, Nantong, China). An equal number of protein (40 μg per lane) were separated by SDS-polyacrylamide gel electrophoresis (PAGE) in 6%, 10% and 12% acrylamide gels and switched to poly vinblastine difluoride (PVDF) membranes (Millipore Corporation, USA) at 300 mA for two hours. The membrane was blocked in 5% fat-free milk and incubated with the following primary antibodies overnight at 4°C: rabbit anti-Rab27a (1:3000 dilution; Abcam), rabbit anti-Bax (1:1000 dilution; Abcam), polygonal rabbit anti-Caspase3 (1:1000 dilution; Abcam), polygonal rabbit anti-Caspase9 (1:1000 dilution; Abcam) and monoclonal rabbit anti-Bcl-2 (1:500 dilution; Abcam). The secondary antibody was horseradish peroxidase-conjugated (HRP)-conjugated goat anti-rabbit antibody (1:1000, Beyotime Institute of Biotechnology). After stripping, the membrane was reproved with β-actin (1:1000, Beyotime Institute of Biotechnology) overnight at 4°C, followed by incubation with secondary antibody as above at room temperature for 2 h. Bands were visualised using an enhanced chemiluminescence system (ECL, Beyotime Institute of Biotechnology). Data were quantified by densitometry.

### shRNA transfection, plasmid constructs and generation of stable cell lines

Three different shRNA sequences targeting human Rab27a and negative control shRNA were designed and obtained from Shanghai Genepharma Corporation. The sequences of Rab27 and control shRNA were as follows: Rab27a shRNA-1 sequence: 5’-CAGTGTACTTTACCAATA-3’, site: 321-338, Rab27a shRNA-2 sequence: 5’-AGGAGAGGTTTCGTAGCT-3’, site: 485-502, Rab27a shRNA-3 sequence: 5’-CTGCCAATGGGACAAACA-3’, site: 743-760, negative control shRNA sequences: 5’-GGATCAGTTAAGTGAAGA-3’, site: 873-890. Cells were then transfected by Lipofectamine™ 2000 (Invitrogen) according to the manufacturer’s instructions. The most effective silencing sequence was filtered by western blot, and then ligated into the PGPH1/GFP/Neo vector. Full-length Rab27a cDNA was cloned into pCMV6/AC/GFP vector (OriGene, USA). Two cell lines were separately transfected with plasmids and selected by GFP sorting. Cells were then grown in complete medium containing 200 μg/ml G418 (Roche Diagnostics, Mannheim, Germany). Fluorescence microscope was utilized to confirm the transfection efficiency. Cells were separated and expanded into clones after four weeks. Subclass cells expressing Neo and Rab27a genes were named as H1650-Rab27a, SPC-A-1-Rab27a. The corresponding controls were named H1650-scr, SPC-A-1-scr.

### Cell proliferation assays

For analysis of cell proliferation, SPC-A-1-Rab27a, H1650-Rab27a, SPC-A-1-scr, H1650-scr, SPC-A-1, H1650 cells and normal control cells (5 × 10^3^) in 100 μL of medium were seeded per well into 96-well plates (three wells each group). Cell proliferation was evaluated using the Cell Counting Kit-8 (CCK-8, Beyotime Institute of Biotechnology) according to the manufacturer’s instructions. Briefly, 10 μL of CCK8 solution was added to the culture medium in each well, and cells were incubated for 3 h. The absorbance was determined at 450 nm wavelength. The assays were repeated three times with triplicate samples.

### Transwell migration and invasion assays

Ambers consisting of Transwell-precoated Matrigel with 8 μm pores were inserted in 24-well tissue culture plates (BD Biosciences, Bedford, MA). Cells from different groups (1 × 10^5^) were plated onto the top chamber in RMPI1640 without FBS and the bottom chamber was filled with RMPI1640 containing 10% FBS as a chemoattractant. After 24 h of incubation in a 5% CO_2_ humidified atmosphere at 37°C, noninvading cells were removed by wiping the upper surface of the membrane with a cotton swab, and the filter membrane was fixed with 4% paraformaldehyde and stained with Exam MaSiLiang blue. The degree of invasion was quantified by counting the cells that had migrated through the membrane in at least six random fields (total magnification, ×200) per filter. Experiments were repeated three times in triplicate. For analysis of cell migration, we use the modified Boyden Chambers without the Transwell-precoated Matrigel membrane filter, using the method performed as above.

### Tumor formation in nude mice

H1650-Rab27a, SPC-A-1-Rab27a and corresponding control cells were injected subcutaneously in mice to investigate the ability to generate xenograft tumours. BALB/cathymic nude mice (4 to 6 weeks old) were purchased from Shanghai Laboratory Animal Center, China and kept in a specific pathogen-free environment. All mouse experiments followed institutional guidelines and were approved by the committee on the Ethics of Animal Experiments of Nantong University, Permit Number: SCXK (hu) 2012-0002. We harvested 1 × 10^7^ cells by incubation in trypsin-EDTA, washed the cells twice with PBS, resuspended the cells in 0.2 mL of RMPI medium, and injected each cell line subcutaneously into BALB/cathymic nude mice. (Eight mice were used per cell line and each mouse received two injections, each of 1 × 10^7^ cells, in the bilateral flank to form two tumors. We injected stably transfected cells into one side of each mouse and the corresponding control cells in the other side.) To explore the effect of altered Rab27a expression on chemotherapeutics, we selected the classical antitumor drug cisplatin. At 7 days of tumor formation, three of the eight mice in each group in which Rab27a was silenced were randomly selected and received an intraperitoneal injection of cisplatin (7.5 mg/kg). The remaining five mice received an injection of the same amount of saline. The tumor growth of different treatment groups was monitored until the day that mice were ended thier life. The date at which the first grossly visible tumor appeared was recorded, and the tumor size was measured every 3 days. Two-dimensional measurements were taken with an electronic caliper after injection, and the tumor volume was calculated with the following formula: tumor volume (in mm^3^) = π/6×a ×b^2^, where a was the longest diameter, and b was the shortest diameter. When a tumor reached 2.0 cm in diameter, the mouse was anesthetized by 1% pentobarbital sodium (Sigma, St. Louis, MO, USA) and photographed. The tumors were excised, weighed and measured. Half of the primary tumors were fixed in 10% formalin overnight and subjected to routine histological examination by investigators who were blinded to the tumor status. The other half was frozen at –80°C for later research.

### Tumor tissue microarray (TMA) and immunohistochemistry (IHC) staining

All tumor samples were embedded in paraffin after fixing in 10% formaldehyde for 24 h and used for constructing the TMA. A representative area of each sample was selected and 2.0 mm tissue cores were designed for constructing a TMA by Shanghai Super Biotek, China. We used hematoxylin-eosin staining (H&E) to confirm the quality of TMA sections. IHC staining was performed as described previously. Briefly, sections (4 μm) were deparaffinized and rehydrated. Antigen retrieval was performed by boiling under pressure in citrate buffer, pH 6.0, for 3 min. Nonspecific binding was blocked by 5% goat serum in PBS for 15 min, and the tissues were incubated with primary antibodies as follows: rabbit anti-Rab27a (1:300 dilution; Abcam), rabbit anti-Bax (1:300 dilution; Abcam), monoclonal rabbit anti-Bcl-2 (1:250 dilution; Abcam), polyclonal rabbit antiCaspase3 (1:300 dilution; Abcam) and monoclonal rabbit anti-Fas (1:250 dilution; Abcam). The secondary antibody was EnVision goat anti-rabbit HRP (DAKO, USA). The immunostained sections were evaluated by two trained pathologists who were unaware of our research purpose.

### Chemotherapeutic cell treatments

Cisplatin (DDP) was used at 0.1–100 μM to determine the half-maximal inhibitory concentration (IC50) values in cell lines in which Rab27a was silenced and their corresponding controls cells. Cells (5 × 10^3^) were added to each well in a 96- well plate and cultured for 24 h. Cells were then treated with drugs for 12 h and the media was replaced by fresh medium without drugs for additional 48 h. Cell viability was measured by a Cell Counting Kit-8 (Beyotime Institute of Biotechnology) at 450 nm as above. DMSO treatment was used as a control. The survival of each cell line was compared with their corresponding control cell line. Assays were repeated three times.

### Statistical analysis

All statistical analyses, including t-test, χ^2^-test, and Mann-Whitney U-test, were carried out with the GraphPad Prism software (version 5; GraphPad Software, La Jolla, CA, USA) and STATA 9.0 software (Stata Corporation, College Station, TX).
